# Targeting Tumors Using Peptides

**DOI:** 10.3390/molecules25040808

**Published:** 2020-02-13

**Authors:** Pablo Scodeller, Eliana K. Asciutto

**Affiliations:** 1 Institute of Biomedicine and Translational Medicine, University of Tartu, Ravila 14B, Tartu 50411, Estonia; 2 School of Science and Technology, National University of San Martin (UNSAM) and CONICET, Campus Migueletes, 25 de Mayo y Francia, San Martín, Buenos Aires CP 1650, Argentina; easciutto@unsam.edu.ar

**Keywords:** phage display, peptides, peptide-drug conjugates, small molecules, tumor associated macrophages, extracellular matrix

## Abstract

To penetrate solid tumors, low molecular weight (Mw < 10 KDa) compounds have an edge over antibodies: their higher penetration because of their small size. Because of the dense stroma and high interstitial fluid pressure of solid tumors, the penetration of higher Mw compounds is unfavored and being small thus becomes an advantage. This review covers a wide range of peptidic ligands—linear, cyclic, macrocyclic and cyclotidic peptides—to target tumors: We describe the main tools to identify peptides experimentally, such as phage display, and the possible chemical modifications to enhance the properties of the identified peptides. We also review in silico identification of peptides and the most salient non-peptidic ligands in clinical stages. We later focus the attention on the current validated ligands available to target different tumor compartments: blood vessels, extracelullar matrix, and tumor associated macrophages. The clinical advances and failures of these ligands and their therapeutic conjugates will be discussed. We aim to present the reader with the state-of-the-art in targeting tumors, by using low Mw molecules, and the tools to identify new ligands.

## 1. The Need for Low Mw Ligands

Low Mw entities offer fundamental advantages over high Mw entities like antibodies or nanoparticles [1,2]. Many tumors like pancreatic and breast dramatically overexpress stromal components, mostly collagen and hyaluronic acid [3,4,5]. This represents a serious barrier that impedes the penetration of large molecules (such as antibodies), because of their lower diffusion coefficient and the high interstitial fluid pressure [6,7,8].

The very high affinity of antibodies is also a limitation to their penetration in tumors, due to a phenomenon known as affinity site barrier [9], which means that the administered antibody binds to the first receptor it encounters and thus cannot explore beyond that. To prevent the affinity binding site barrier, the binding constant K_D_ should not be lower than 10^−9^M, since tumor accumulation does not increase beyond that K_D_ [9].

If we take into account that in a tumor the number of receptors available to peptides from the circulation is normally low (picomoles per gram of tumor [10]), we need to consider using a potent therapeutic/diagnostic companion for the targeting ligand.

Targeted therapies must consider both tumor heterogeneity and inter-patient heterogeneity. In designing mouse-based preclinical studies, the targeting ligand should be chosen based on the tumor type and a marker consensuated to be over-expressed in that cancer in mice and humans. For clinical studies, the over-expression of the marker intended to target should be confirmed for each patient individually, by biopsies and immunohistochemical detection.

## 2. Identification of Peptides by Biopanning

Peptide binders can be identified using peptide libraries displayed on bacteriophage (hereafter referred to as “phage”). In 1985, Smith showed that fusion proteins can be expressed on the capsid of phage virus by introducing foreign DNA material in the phage [11]. This opened the door to biopanning studies to screen and select for binding peptides [12] and also antibodies [13].

Initially, the peptide phage libraries consisted of a linear chain of 6 variable aminoacid residues, X_6_ (where X denotes a random aminoacid) [14]. Later, the random sequence was inserted between two cysteines to obtain cysteine-to-cysteine disulfide cyclized peptides to reduce conformers and improve affinity. The first cysteine-bridged peptide phage library, developed in 1992 by DeGrado and co-workers [15], was of the type CX_6_C and was displayed on the filamentous bacteriophage M13. Because random stop codons occur, this library also gives rise to the linear peptides CX_6_ or shorter. In the T7 bacteriophage system, the variable foreign peptide is inserted at the C-terminus of the coat protein 10A, while in the filamentous M13 bacteriophage, the insert is placed on the N-terminus of the protein III [16].

In 1996, Ruoslahti and Pasqualini introduced in vivo biopanning using peptide phage display [17] and showed that each organ has a different vascular “zip code” that can be probed with the phage library. In in vivo tumor biopanning, the phage library in PBS is injected into a tumor-bearing mouse, allowed to circulate, and is later followed by perfusion to remove unbound and weakly bound phage. The phage which remain in the tissue after perfusion are the strongest binders and are easily amplified by incubating a lysate of the tumor with a suspension containing the bacterial phage host.

Multiple rounds of biopanning are carried out in this way to enrich in the strong binders. This methodology, which is described in fine detail elsewhere [18] has yielded a large collection of tumor-homing peptides to date [19], and we have also used it to screen mice with Alzheimer’s disease [20] or penetrating brain injuries [21].

In vivo biopanning has also been performed in humans, in terminal cancer patients [22]. In vivo phage display from biopsied human tissue was pioneered by Pasqualini and coworkers in 2002 on a patient with B cell malignancy refractory to chemotherapy and with infiltration of tumor cells in the bone marrow [22]. The authors used a CX_7_C library expressed on M13 phage and intravenously infused the patient with the phage library dispersed in 100 mL of saline, and after 15 min of circulation, the tissues were biopsied, the phage were recovered and the sequences analyzed. The authors recovered a phage clone (sequence: CGRRAGGSC) that bound to interleukin 11 receptor alpha (IL-11Rα) which was overexpressed in the prostate. In subsequent studies, this clone was shown to bind to human samples of prostate cancer from 90 patients [23] and to home to IL-11Rα in prostate tumors in mice [24]. The sequence was validated in vitro as a peptide when the authors showed the cytotoxicity to cultured prostate cancer cells of the peptide conjugated to a proapoptotic moiety [23]. In a subsequent in vivo biopanning study in humans in 2011 [25], Pasqualini, Arap and coworkers used the pool recovered from the patient of 2002 for two further rounds of in vivo selection, serially, in two patients with metastatic prostate cancer. Using high throughput sequencing they analyzed 2.4 × 10^6^ tripeptide motifs recovered from the biopsied tissues and found that the organs had preferences for particular motifs. There was no mention as to whether the original isolated clone CGRRAGGSC was enriched during these additional two rounds. Intriguingly, there was also no mention to the presence of the known integrin-targeting tripeptide RGD.

### 2.1. Enhancement of Identified Binding Motifs

Short (defined here as being ≤ 10 residues) linear peptides generally possess a low binding affinity, given their high conformational freedom, and they also display poor resistance to proteolytic degradation. However, it is possible to add modifications to the identified binding sequence in order to circumvent both of these problems.

Cyclization of a linear sequence constrains the peptide and can increase the affinity [14,26,27]. A simple way to achieve cyclization is through the addition of N-terminal and C-terminal cysteines that are intentionally oxidized during the peptide synthesis to form a disulfide bond. An alternative to forming a loop between the ends of the peptide is to perform N-terminal to C-terminal cyclization, also known as backbone or head-to-tail cyclization. This not only constrains the peptide but also provides a higher resistance to proteases [28,29].

The term stapling refers to forming a covalently linked hydrocarbon bridge between two non-natural O-allylserine residues inserted in the peptide [30]. Stapling is intended to increase the rigidity of an α helix in a peptide, hence the linkage must be spaced apart in a way that it does not disturb the twist of the helix. Stapling reduces the number of conformers, increases serum stability and in some cases improves cell permeability, depending on the hydrophobicity of the peptide and the staple [31]. Nuclear magnetic resonance (NMR) spectroscopy, a powerful experimental tool to assess the structure and conformation of proteins and peptides in solution, can be used to corroborate the stabilization generated by the staple [30]. The company Aileron Therapeutics (Watertown, MA, USA) has developed a stapled, cell-permeable, alpha helical peptide, called ALRN-6924, which blocks the tumor-promoting interaction that occurs between p53 and its endogenous inhibitor. The company has an ongoing clinical trial with ALRN-6924 alone or in combination with the chemotherapeutic drug cytarabine, for acute myeloid leukemia (clinical trial identifier: NCT02909972).

The binding of low affinity ligands can also be increased by increasing their avidity, i.e., presenting the ligands in a multivalent manner, as in dimers, trimers [32] or multibranched star shapes [33]. In this approach the binding constant of the multivalent ligand scales with the number of valencies [34].

An appealing and nature-inspired approach to increase affinity, stability and provide oral activity, is to graft the binding motif to natural peptidic structures known as cyclotides. Cyclotides are naturally occurring peptides in plants that were discovered by Craik in the 1990s [35]. These peptides are head-to-tail cyclized and are additionally stabilized by disulfide bonds between non contiguous cysteines. This particular structure endows them with an unprecedented rigidity and makes them resistant to proteases and high temperatures [36,37,38,39]. An example of a cyclotide is the sunflower trypsin inhibitor-1 (SFTI-1), a 14 aminoacid residue peptide consisting of two loops separated by one disulfide bond. One of its loops inhibits the activity of trypsin and pepsin, thus further increasing the endurance of the peptide to proteases and rendering it orally active [40,41]. It has been reported that known binding motifs can be grafted to the second loop of SFTI-1 amplifying the affinity of the motifs for their receptors while preserving the favorable features of the parental SFTI-1 [36,41,42]. Another cyclotide, called Kalata B1, derived from the kalata plant, presents a structure of 29 aminoacid residues knotted by three disulfide bridges [35]. Kalata B1 is resistant to high temperatures and to the conditions found in the gastrointestinal tract. Infusions of the kalata plant are used in the congo region in Africa by pregnant women to induce labour, because of the ability of Kalata B1 to induce uterine contractions.

Peptidic ligands that do not withstand the gastrointestinal tract need to be administered in the blood and generally present a fast renal clearance because of their low molecular weight. A short blood half-life is an advantage for radioligands, which are used in radiotherapy to concentrate alpha or beta radiation in the tumor [43] but induce unwanted damage while they circulate.

The blood half-life of a peptide can be extended by conjugating it to a higher Mw long-circulating molecule, such as polyethylene glycol (PEG) [44,45]. The tumor penetration, in lymphoma-bearing mice, of a tumor-targeting peptide [46] conjugated to PEGs of different Mw (ranging from 40 KDa to 150 KDa) scaled linearly with the Mw of PEG [45], despite the higher Mw and hence slower diffusion. However, the authors did not include the non PEGylated peptide in their study and lymphoma is not stroma-dense, so the advantages of PEGylating a peptide for targeting solid tumors with aberrant stroma remain to be seen.

A widely used approach to extend a peptide’s half life exploits the long-circulating capacity of serum albumin and relies on coupling an albumin-binding moiety to the peptide, such as Evans blue [47,48], fatty acids [49,50], short albumin-binding peptides [51], or albumin-reacting moieties like an extra cysteine [52] or a maleimide group [48] to form a disulfide bond with albumin-Cys34. Another strategy relies on coupling to the peptide a small molecule for binding to another long-lived serum protein, transthyretin [53]. However, it must be noted that by associating the peptide with a long-circulating molecule so that the Mw of the ensemble is above renal cutoff, the ensemble is no longer a low Mw entity.

### 2.2. Alternative Biopanning Strategies

More sophisticated biopanning strategies have been devised to identify peptides with higher affinity and stability. To address the issue of the poor proteolytic stability of peptidic ligands, in 1996, Schumacher and co-workers proposed a method of identifying d-peptidic binders, since d-aminoacids are not recognized by mammalian proteases. Their elegant approach, called mirror image phage display [54], consisted of screening a d-aminoacids containing protein of interest with a library of natural l-peptides. The d-enantiomer of the identified l-peptide binder was then shown to bind to the natural l-enantiomer protein. The drawback of this approach is that because it is performed on d-proteins, it cannot be carried out in vivo.

After the initial linear and disulfide-cyclized peptide libraries, new strategies appeared to identify constrained peptides with higher affinity for their targets. The first of such developments was introduced by Heinis, Winter and co-workers in 2009, with bi-cyclic peptide libraries [55]. In this filamentous bacteriophage library, the random chain of aminoacids contains three cysteines, two in the ends and one in the middle. The phage library is then chemically modified to link the three cysteines with a three-armed linker [tris-(bromomethyl)benzene] to form a macrocycle that contains two loops. In the first proof of concept, this approach was used to identify a low nM affinity ligand to kallikrein [55], an enzyme implicated in human cancer [56]. Although oral activity has not yet been reported, these high affinity bicyclic peptides have enormous pharmacological potential, because of their selectivity and affinity to the receptor and a superior tumor penetration compared to antibodies. This technology has spawned the company Bicycle Therapeutics (Cambridge, U.K.) which uses this screening platform to develop new drugs.

## 3. In Silico Identification of Peptides

Biopanning is not the only path to identify a good peptidic ligand. Several peptides have been identified in silico using random generation algorithms, modification of existing databases or conceived by imitating protein fragments known to bind a desired receptor.

Crude random generations algorithms are the simplest methods in which, given a desired length for a peptide ligand, all the resulting possible combinations for sequence and structure are tested. The number of possible sequence combinations for a peptide with length n is 20^n^; and for each combination, an extensive number of possible structural conformations exist, increasing enormously the number of peptide conformations. Increasing the length of the peptide, n, not only increases the number of possible combinations of amino acids, but also the number of possible peptide bond torsions, that is, increases the number of degrees of freedom of the system. It is evident that a random generated library can be extremely computationally demanding, even for very small peptides. However, using this technique, Panyayai and coworkers reported WCW as the tripeptide with best inhibitory activity to angiotensin-I converting enzyme (ACE) [57], an enzyme with tumor-promoting roles [58], and the inhibition of which shows reduced mortality and recurrence in many cancer types [59]. In their work, they started with a random library of 8000 tripeptides generated using a python algorithm. This library was docked to the ACE structure and the 600 best scoring compounds were selected. After an analysis of site occupancy and physical properties of each candidate they finally selected five tripeptides to test experimentally, and WCW resulted as the best candidate showing an IC50 value of 49 µM. A similar procedure was applied by Mollica and coworkers to discover novel tripeptides with the ability of inhibiting human α-glucosidase enzyme [60]. The authors generated a library with all combinatorial possibilities for a tripeptide and then followed a process of docking, molecular mechanics/generalized Born surface area (MM/GBSA) refinement, and molecular dynamics using several components of the Schrödinger package [61,62,63]. The resulting four peptides from this method inhibited α-glucosidase with an inhibitory potency equivalent to the one associated with the peptide used as template, acarbose.

In the mentioned works, the number of initial candidates generated by the random algorithm were reduced using mainly physical and chemical principles. A different approach was undertaken by Michaeli and coworkers [64]. They used the risk-adjusted design algorithm [65], a finance-based algorithm, to computationally design linear and cyclic peptides targeting proteins MD2 and CD14, both co-receptors of human toll-like receptor 4, which is associated with chemoresistance in breast cancer [66]. The algorithm reduces the number of candidates by assigning them a “reward” or a “risk”. The rewards are calculated using an energy binding approximation, while the risk is related with possible steric clashes that could occur as a result of a particular substitution. With this algorithm, the authors could predict a total of 26 cyclic and 27 linear peptides that bind to the human TLR4 co-receptor complexes.

Another strategy of in silico peptide design is to use a pre-existing database instead of generating one. A good example of this approach is that used by Vukic et al., identifying a set of milk derived di- and tri-peptides with high antihypertensive activity for the angiotensin-I-converting enzyme [67]. They used an ACE-inhibiting milk peptide database, containing aminoacids sequences and activities, and applied the CoMFA model [68,69] combined with molecular docking techniques to do the search. CoMFA model is based on a 3D quantitative structure activity relationship (3D-QSAR) [70,71] that employs both interactive graphics and statistical tools for correlating geometries and physical properties of molecules with their biological activity. Although 3D-QSAR models for ligand predictions have been used for more than three decades now, its use with peptides is very recent, since older models were not capable of dealing with the peptide’s high conformational flexibility.

A useful in silico peptide design strategy consists of simulating protein fragments known to bind a desired receptor. A good example of this methodology is the prediction and design of two peptides, P109 and P110 based on a thorough study of the interactions between the NTF2-like domain of G3BP (overexpressed in human tumors [72]) and the SH3 domain of RasGAP [73]. Another example is the work by Geng et al. The authors designed two 27-mer peptides to target the extracellular domain of HER2 protein using a combination protocol of molecular dynamics, MM/GBSA binding free energy calculations, and binding free energy decomposition analysis [74]. The design was based on the known interactions between a previously reported affibody to HER2 (ZHER2:342) [75] and HER2 and a later rational structural modification. Basically, important identified binding residues were kept and linked together with glycine residues according to previously calculated distances.

### Protein-Peptide Docking Methods

Success of in silico virtual peptide library screening strongly depends on the efficiency of the docking techniques employed to select the best candidates. Current peptide docking techniques can be divided into two categories: *local peptide docking*, wherein information of the binding pocket is available and docking focuses on finding the binding modes of a given peptide at the known binding pocket/site of a target macromolecule [76,77,78]; and *global peptide docking,* in which no information on the binding site is available. Local peptide docking is challenging due to the high flexibility of peptides, and consequently large number of possible values for the several torsion angles between peptide bonds, but the real challenge occurs in *global peptide docking*, where no information regarding the location of the binding region is available, and thus, as the name implies, the search has to be global, around the entire surface of the macromolecule. This type of search is referred to as ’blind docking’. Very few works deal with peptide blind docking, in which a flexible peptide is docked to a target macromolecule without prior knowledge of the binding site.

The first successful study was performed by Hetenyi and coworkers using the Autodock package for blind docking of di- and tri-peptides [79]. The method was powerful in locating the binding pockets and binding orientations for protein-small peptides complexes.

For medium size peptides (up to nine residues) successful attempts of blind docking were achieved by some of the second generation docking techniques (called “ensemble docking”) [80,81,82]. One of them, HPEPDOCK81, treats the peptide flexibility using a hierarchical algorithm that models peptide conformations and progressive global sampling of binding orientations. Using HPEPDOCK, Srinivasan et al. designed de-novo tripeptides that bound to a mutant form of the copper/zinc dismutase SOD1 [83], an enzyme which has been assigned pro-tumoral roles [84].

The latest generation of docking methods includes full peptide flexibility, sampled at the same time as the global protein search is performed. These methods are called *de-novo* docking methods. Some of them also have the ability of making blind docking. One of these *de-novo* docking methods, AnchorDock [85], restricts the docking search to the most relevant parts of the conformational space. Preliminary restrictions are performed by identifying anchoring spots on the protein surface in a first step, and then performing simulated annealing molecular dynamics around the predicted protein anchoring spots.

Recently, Kurcinski et al. developed a web server interface (The CABS-dock protocol) that performs *de novo* blind docking for 5–15 aminoacid peptides [86,87]. The method also allows to exclude domains in the macromolecule that are known to not participate in the studied docking problem with the aim of reducing conformational space search. An advantage of this protocol is that it is very user friendly, and only requires as inputs a PDB for the receptor and the sequence of the peptide. To improve the quality of the method some extensions were added, as a refinement step using molecular dynamics and the possibility of incorporation of experimental data [87]. Zhang [88] and coworkers developed a new version of Autodock, also oriented to *de novo* peptide blind docking; the software is called AutoDock CrankPeP (ADCP). It makes use of CRANKITE [89], a software package that samples the conformational space of proteins using a Metropolis Monte Carlo method. In ADCP, CRANKITE is combined with the grid-based AutoDock representation of a rigid receptor to optimize at the same time peptide conformations and peptide-receptor interactions. ADCP showed a success rate greater than 85% on the LEADS-PEP dataset when considering the top 10 predictions. It is also able to dock peptides with up to 20 amino acids to their receptors.

De novo methods are currently the most accurate ones for peptide blind docking or binding pocket prediction, as are also less affected by the peptide’s length. A particular challenging case appears when the binding site is located at an interface. To address this problem there exist a variety of tools as CASTp [90], MAPPIS [91], MolSurfer [92], and ProFace [93], all designed to find residues at interfaces that show strong interactions between proteins. Regarding peptides, Wang and coworkers [94] found short peptides that target programmed cell death protein 1 (PD-1) to inhibit its binding to programmed cell death ligand 1 (PD-L1). The unknown binding pocket of PD-1 is in fact part of PD1/PD-L1 interface. The authors used all of the four mentioned packages: CASTp, MAPPIS, MolSurfer, and ProFace and selected the residues that were common findings between all the methods as residues defining the binding site based on interactions. As a result, peptides that bind to the PD-1 receptor with moderate affinity were found confirming, at least in part, that the proposed binding pocket was correct.

The virtual screening methods discussed so far make use of different structure determination and docking protocols, but not all of them are freely available. Ansar and coworkers [95] developed a graphical user interface-based pipeline that integrates different existent tools for performing the complete process of virtual screening of peptides. The pipeline is called PepVis, and it features both ensemble and flexible de novo docking protocols. The tools included in PepVis are freely available tools and show a good performance in benchmarking of protein–peptide docking studies (i.e., ModPep [96] + Vina [97]). The docking protocols we have exposed are summarized in Table 1.

## 4. Vascular-Targeting Peptides

Perhaps the most paradigmatic tumor-homing peptide motif is RGD, which targets integrins over-expressed on tumor blood vessels. After identifying fibronectin in 1973 [98], Ruoslahti and collaborators, in 1984, discovered that a small fragment in fibronectin, the RGD motif, was responsible for its binding to cells [99]. In 1985, Ruoslahti and co-workers identified the receptor for RGD, using affinity chromatography, from an extract of osteosarcoma cells [100]. The receptor was a 140 KDa cell surface glycoprotein. That protein identified by Ruoslahti was an integrin, although the name integrin was only introduced a year later by Hynes when he identified another member of the integrin family [101]. The RGD motif was later shown to bind to different members of the integrin family [102].

Interestingly, when biopanning was performed on recombinant α_5_β_1_ integrin using a linear X_6_ library, the vast majority of the selected binders contained RGD, and one peptide where RGD was contained between two cysteines (presumed to be cyclized) showed stronger binding than the linear ones [14].

A tumor-penetrating version of the RGD peptide was identified in 2009 (denoted iRGD, “i“ for internalizing) by in vitro biopanning of the CX_7_C library on prostate cancer cells [103]. The ligand iRGD contains, besides the integrin-binding motif, a C-terminal sequence that binds to neuropilin-1 (NRP-1). The NRP-1-binding motif needs to be on the C-terminus for binding to occur, and this condition was termed the “C-end rule” or CendR. It was possible to encounter this condition because the T7 bacteriophage was used for the screens, where the insert is on the C-terminus of the coat protein. The NRP-1-binding motif is only exposed after proteolytic cleavage, something which is favored in the tumor microenvironment. Binding to NRP-1 triggers tissue penetration of the peptide and drugs administered with it [104,105]. The discoverers of iRGD also reported anti-metastatic properties of the peptide in mouse models of pancreatic and prostate cancer, and the effect was mediated by the binding to NRP-1 [106]. Different research groups over the last 10 years have shown that iRGD enhances drug accumulation and efficacy in a number of tumor models [107,108,109,110,111], and we showed that iRGD also enhances the tumor accumulation of co-administered chemotherapy when it is given intraperitoneally to treat metastases in the peritoneum [112]. A company called DrugCendR (La Jolla, CA, USA) was founded by Ruoslahti to translate the oncological applications of iRGD, and in 2018 iRGD entered phase I clinical trials as an additive to gemcitabine and abraxane for pancreatic cancer (clinical trial identifier: NCT03517176).

**Figure 1:**
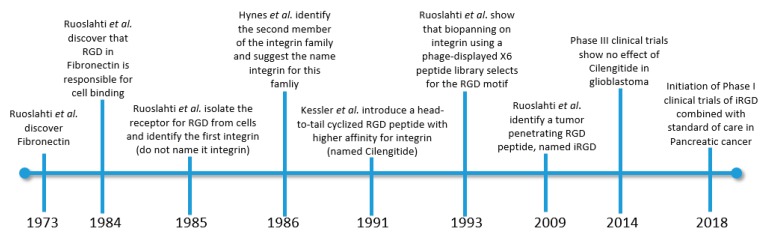
Milestones in the genesis and evolution of RGD in tumor targeting.

A head-to-tail cyclized version of RGD was developed by Kessler and collaborators in 1991 [27,113]. The authors introduced a d-aminoacid that induced a defined conformation and this facilitated head-to-tail cyclization. It was the cyclization and not the introduction of additional D-aminoacids that potently increased the binding activity of this cyclic RGD. The most used and studied variant of the cyclized RGD is denoted cyclo(RGDfV), where f is d-phenylalanine and V is valine. The peptide cyclo(RGDfV) was later commercialized for research use under the name cilengitide. Cilengitide alone proved to be effective in reducing glioblastoma in mice [114] and also in phase I and II clinical trials [115], presumably because of its anti-angiogenic effects [113]. However, in phase III clinical trials cilengitide did not show an effect on survival [116] and its development was halted. The reasons for the failure of this phase III were suggested to be related to the poor pharmacokinetics of this small peptide (which was given intravenously) and an ineffective administration regime [117]. The milestones in the genesis and evolution of RGD in tumor targeting are schematized in Figure 1.

Recently, an RGD-containing bicyclic peptide was identified by screening a library of 672 synthesized macrocyclic peptides that contained RGD and differed in their cyclization structure. The strongest binder showed a striking 0.4 nM affinity towards α_ν_β_3_ integrin. The authors showed in vitro binding to receptor-positive cells although information on the peptide’s biological effect is still unavailable [118].

Haberkorn et al. have identified an α_ν_β_6_ binding peptide by screening a phage library based on a sunflower trypsin inhibitor-1 scaffold [119]. The selected peptide included the motif FRGDLMQL and showed strong binding to α_ν_β_6_ (K_D_ ≈ 15 nM). The peptide was used as a radioligand, coupled to a PET-active probe (^68^Ga) in tandem with the radiotherapeutic agent ^177^Lu. This peptide-drug conjugate showed selective tumor accumulation in patients with head and neck squamous carcinoma and non-small cell lung cancer, as evidenced through PET imaging, forecasting potential therapeutic success.

Del Gatto et al. have rationally designed an RGD-containing peptide that binds to α_ν_β_3_ integrin and not to α_ν_β_5,_ by using the X-ray structure of α_ν_β_3_ complexed with cilengitide and the NMR structure of the disintegrin Echistatin; they named this new peptide RGDechi [120]. RGDechi conjugated to a PET imaging probe homed to α_ν_β_3_+ tumors and not to α_ν_β_5_+ tumors [121]. More recently, the same group mapped the interactions between RGDechi and α_ν_β_3_ by performing NMR spectroscopy of RGDechi and a α_ν_β_3_-containing cell membrane, and molecular docking studies of this complex [122]. The ability to target α_ν_β_3_ integrin is important because of its role in tumor angiogenesis and metastasis [123,124].

Stallcup, Ruoslahti, and colleages, identified in 1999 a peptide that binds to the chondroitin sulfate proteoglycan NG2 [125], an attractive target, as it is expressed on angiogenic blood vessels in tumors. In vitro biopanning was carried out using a 10-mer linear phage library on wells coated with recombinant NG2 and after several rounds the peptides TAASGVRSMH and LTLRWVGLMS were enriched. The two phage clones containing those inserts homed to NG2+ tumor vasculature, but not to NG2 knockout tumors; however, the selected motifs were not tested as free peptides. In a 2014 study, the TAASGVRSMH peptide was conjugated to docetaxel-loaded nanoparticles; this guided the nanoparticles to NG2+ vascular pericytes in the tumor and enhanced their efficacy in a model of metastatic melanoma [126].

Another tripeptide motif with well validated tumor vasculature homing is NGR [127]. An NGR-containing phage clone was first isolated by Koivunen and Ruoslahti during in vitro biopanning on α_5_β_1_ integrin [14]. The NGR motif binds to integrins with low affinity, but its receptor is Aminopeptidase N (APN/CD13) [128], a protein which is detectable in tumor vasculature and in angiogenic blood vessels. A disulfide-cyclized peptide containing the NGR motif, CNGRC, conjugated to doxorubicin, was toxic to APN-transfected cancer cells in vitro and not to the non-transfected controls [128].

The vascular-homing peptides described here are summarized in Table 2.

## 5. Extracellular Matrix-Targeting Peptides

Almost all solid tumors overexpress extracellular matrix (ECM). The ECM components which are over-expressed include collagen, connective tissue growth factor, versican, chondroitin sulfate proteoglycans [129,130,131] and isoforms and alternatively spliced forms of tenascin-C [132] and fibronectin extra domain B [133].

In 2017 we screened mice with Alzheimer’s disease using a CX_7_C peptide T7 phage library and collected a peptide named “DAG” (disulfide bonded CDAGRKQKC) that targets connective tissue growth factor (CTGF/CCN2) [20]. The applicability of the DAG peptide was demonstrated for Alzheimer’s disease, as we found that CTGF is overexpressed in early and late stages of the disease in mice and also in humans [20]. Besides its presence in the ECM, CTGF is also expressed on endothelial cells, in cancer [134] and in early Alzheimer’s disease, thus DAG should be considered both an ECM and a vascular targeting ligand.DAG showed high accumulation in a patient-derived xenograft of glioblastoma, binding to activated astrocytes and extracellular matrix in the tumor. The matricellular protein CTGF is known to be upregulated in several glioblastoma models in mice [135] and also in human brain tumors [136], it is mainly produced by activated astrocytes and represents an attractive target for the treatment of glioblastoma [137].

More recently, a peptide (dubbed PL1) that binds to fibronectin extra domain B (EDB-FN) and tenascin-C was identified by performing in vitro biopanning using a CX_7_C phage peptide library on both recombinant proteins [138]. PL1 (sequence: PPRRGLIKLKTS)-conjugated nanoparticles targeted several different intracraneal glioblastomas in mice, which expressed one or both receptors, which are often over expressed also in human brain tumors. In vivo validation of PL1 as a free peptide has not yet been published.

Lu and collaborators identified in 2015 another peptide that binds to EDB-FN [139]. The peptide, termed ZD2 (disulfide-cyclized CTVRTSADC), has an affinity of 11 µM for EDB-FN and was identified by screening a CX_7_C commercial M13 phage library on recombinant EDB immobilized on plates. Conjugates of ZD2 and a ^68^Ga chelate or Gd-DOTA, are currently being developed by the company Molecular Theranostics (Cleveland, OH, USA) as contrast agents for PET and MRI imaging, respectively, for prostate cancer.

Hamzah and colleagues have attacked the problem of targeting tumor ECM in a clever way [140]. Using a CX_7_C library, they performed in vitro biopanning on Matrigel^TM^. The selected pool after three in vitro rounds was then administered in breast tumor-bearing mice for in vivo biopanning. Four rounds of biopanning yielded the peptide CSGRRSSKC (named CSG). Disulfide cyclized CSG displayed tumor homing in different mouse models and was shown to bind to laminin-nidogen complexes. By performing the phage selection on Matrigel^TM^, the authors were able to identify a peptide that binds to an ECM target that is not associated with blood vessels or in the vicinity of the vasculature. It remains to be seen if the peptide requires a leaky tumor vasculature in order to reach this “meta-vascular” target. Leaky vasculature is a phenomenon that occurs homogeneously in tumors xenografted in mice but is a heterogeneous phenomenon in human tumors [141,142].

Hubbell and co-workers have developed extracellular matrix-binding peptides with exciting applications in cancer therapy by imitating natural ligands [143,144,145,146]. An ECM-binding peptide derived from the placenta growth factor (PlGF-2), dubbed PlGF-2_122–144_ (sequence: RRRPKGRGKRRREKQRPTDCHL) bound to fibronectin, fibrinogen, osteopontin, vitronectin, and collagens I, II, III and IV with a KD in the low nM range for fibronectin I and collagen I. The peptide was coupled to the immune checkpoint inhibitors anti-CTLA4 and anti-PD-L1, which enhanced their tumor retention and increased their therapeutic effect [147].

Bicycle Therapeutics has developed a peptide-drug conjugate (named BT1718) that targets matrix metalloproteinase-14 (MMP-14), an enzyme that is over-expressed in certain solid tumors [148]. BT1718 consists of a bicyclic ligand coupled to maytansinoid (a microtubule polymerization inhibitor) through a disulfide cleavable linker. BT1718 binds with high affinity (K_D_ ≈ 1.5 nM) to MMP-14 and Phase I/IIa trials are underway in patients with advanced solid tumors (triple negative breast cancer and non-small cell lung cancer) (clinical trials identifier: NCT03486730). The ECM-homing peptides described here are summarized in Table 3.

## 6. Tumor Associated Macrophage-Targeting Ligands

Anti-inflammatory or M2-skewed tumor associated macrophages (M2 TAMs) are main effectors of metastasis, angiogenesis, and immunosuppression [149,150]. They derive from circulating monocytes and are skewed to the M2 phenotype by tumor-secreted cytokines, like interleukin-4 (IL-4) and macrophage-colony stimulating factor (M-CSF). M2 TAMs navigate through the collagen-dense tumor matrix aided by a palette of collagen-interacting proteins such as matrix metalloprotease MMP-9 [151], collagenases [152] and the collagen-endocyting receptor CD206 (mannose receptor, CD206/MRC1). M2 TAMs wander through the ECM forming new blood vessels by secreting VEGF [153] and opening short-lived openings in blood vessels for tumor cells to intravasate [154]. Due to these traits, this subpopulation of macrophages represents a major therapeutic target, and large efforts have been placed in recent years to identify ligands to M2 TAMs.

The company Riptide Biosciences (Vallejo, CA, USA) designed a linear peptide that binds to CD206. The peptide, named RP-182 (sequence: KFRKAFKRFF) consists of alternating hydrophobic and hydrophilic aminoacids. Because the peptide also binds to RelB and CD47, the selectivity of systemically administered RP-182 for M2 TAMs remains to be seen. Interestingly, in a congress abstract [155] the peptide was recently reported to induce phagocytosis and apoptosis in M2 mouse macrophages. This activity could be linked to the conformational change of CD206 produced by RP-182.

Another peptide, named M2Pep (sequence: YEQDPWGVKWWY) was identified in 2013 by Pun and collaborators by performing in vitro biopanning on M2-skewed mouse macrophages [156]. M2pep was coupled to a pro-apoptotic peptide and administered to subcutaneous colon tumor-bearing mice. The treatment was able to extend the survival respect to the untreated mice and suggested that the observed M2pep-targeted TAM depletion was the cause for the enhanced therapeutic benefit. To increase the binding of M2pep to M2 macrophages through avidity, the same authors then synthesized a di-, tri- and tetravalent-peptide conjugate, by coupling M2pep with an added C-terminal cysteine, to maleimide groups on a multi-maleimide scaffold [32]. The tetravalent version had a higher M2/M1 selectivity than its monovalent counterpart, and when conjugated to the pro-apoptotic peptide, the toxicity towards M2 macrophages was augmented in the tetravalent version respect to the monovalent one. In a follow-up study, Pun and co-workers enhanced M2pep by introducing several modifications [26]. First, they disulfide-cyclized it by inserting terminal cysteines. They also noted a rapid cleavage in serum of the bond between tryptophan W10 and W11 of M2pep. Therefore, they substituted W10 for a tyrosine; this increased the serum stability and as a consequence improved the binding. They showed that both disulfide cyclization and substitution of WW for YW resulted in a higher affinity towards M2 macrophages in vitro. The authors also added a three d-lysines (kkk) spacer at the C-terminus to improve the serum stability, although no rationale was provided for this modification. Despite the facts that the receptor for M2pep and cyclicM2pep(RY) have not been determined, that they are hydrophobic and must be dissolved in 10% DMSO, and that binding to human M2 macrophages has not been shown, these two peptides represent promising M2 TAM targeting ligands, as demonstrated by their high in vitro selectivity.

We identified in 2017 a CD206-binding peptide by performing phage display on mice with metastatic breast cancer [157]. The peptide, termed UNO (disulfide cyclized CSPGAKVRC), homed and internalized in M2 TAMs of solid tumors of different models [157]. We further identified the minimal binding motif of UNO to CD206 to be CSPGAK (termed mUNO), which interestingly holds a sequence that is present in all CD206-binding collagens. The peptide mUNO also showed homing to M2 TAMs. We recently showed binding of mUNO to human recombinant CD206 and selectivity of the peptide for human M2 macrophages derived from primary monocytes. Modeling studies of mUNO and human CD206 showed that the peptide interacts with a binding pocket on CD206 to which no other ligand has been reported [158]. This binding pocket lies between C-type lectin domain 1 and C-type lectin domain 2. Modeling and also experimental binding studies showed that mUNO and mannose do not compete for the same binding site. This renders mUNO more specific than mannose-based ligands, which bind to other mannose-binding proteins, like CD209 [159,160]. The M2 TAM-targeting agents described in this review are summarized in Table 4.

## 7. Non-Peptidic Ligands

Low and colleagues reported in 1991 that macromolecules could be shuttled inside cells using the folic acid receptor [162]. This small molecule, also known as vitamin B9, has since been used as a drug delivery vehicle, coupled to macromolecules, liposomes, radioactive imaging agents, chemotherapy drugs and other small molecules [163,164,165]. The folate receptor is reported to be overexpressed in approximately 40% of human cancers. Two different isoforms of the folate receptor are overexpressed in tumors, and they both bind folic acid with high (nM) affinity. The folate receptor-α is overexpressed on cancer cells and the folate receptor-β isoform is expressed on M2 tumor associated macrophages [166].

A company co-founded by Low, called Endocyte (West Lafayette, IN, USA), performed clinical trials using folate to steer the tubulin-disruptive drug vinblastine into tumors. The folate-vinblastine conjugate, called vintafolide, reached phase III in clinical trials in platinum-resistant ovarian cancer. However, in 2014 the phase III was stopped because vintafolide was unable to increase the progression free survival. A later study [167], found high levels of the multidrug resistance protein P-glycoprotein (PgP) in those patients who were positive for folate receptor but did not respond to the treatment, and the study recommended to exclude patients whose tumors are PgP^high^ from future clinical trials.

A clever immunotherapeutic application of folate targeting has emerged last year from the Low laboratory. In this approach, folate is used to target fluorescein to tumors, and anti-fluorescein-engineered chimeric antigen receptor-T (CAR-T) cells are then administered to the mice. This methodology proved highly effective in treating different tumors grown in mice [168,169].

Small molecule phosphoramidate compounds have been developed to target the prostate specific membrane antigen (PSMA), which is overexpressed in human prostate cancers [170,171]. These consist of a family of compounds with varying affinities, some of which irreversibly bind PSMA [172]. These PSMA binders have been used as radioligands to transport the beta-emitting radioisotope ^177^Lu, through a dodecanetetraacetic acid (DOTA) moiety conjugated to the ligand. The Novartis company (Basel, Switzerland) has an ongoing phase III clinical trial to treat castration-resistant prostate cancer with tumor targeted radiotherapy, by using 177Lu-PSMA (clinical trial identifier: NCT03511664).

Mannose-containing molecules (mannose Mw: 180Da) can be used for cancer theranostics and vaccines to target the mannose receptor MRC1/CD206 which is overexpressed on pro-tumoral macrophages and also present on dendritic cells. Coating nanoparticles with mannose can increase their uptake in M2 macrophages [161]. In vivo, a few studies claim to target payload to M2 TAM by using mannose, however they do not show if the enhanced tumor accumulation observed is due to M2 TAM capture [173], and other studies do not show the results with the non-mannose-targeted control [174].

Although not a low Mw molecule, the multivalent mannose ligand, Manocept, developed by the company Navidea is worth mentioning here. Manocept coupled to a ^99^technetium-chelating agent is called γ-tilmanocept, it has a Mw of 18 KDa and an extremely high affinity (K_D_: 30 pM) for CD206 [175]. In 2013, the FDA approved the local administration of tilmanocept (with the commercial name of Lymphoseek) as a contrast agent to detect CD206^+^ macrophages in the lymph node, shown to be a marker of early head and neck and breast cancer [176]. Mannose has also been used to guide polymers to dendritic cells for cancer vaccines [177,178]. As other mannose-binding proteins, like CD209 (DC-SIGN) are highly expressed in the intestinal and skin tissue, mannose is expected to have off-target effects when administered sistemically.

## 8. Concluding Remarks

We have presented here a number of different techniques for identifying peptidic ligands, to target cancer for therapeutic or diagnostic applications, each of which have advantages and disadvantages, as summarized in Table 5. The oldest and most validated approach is to use phage-displayed peptide libraries of the sort CX_i_C. Since this yields ligands with low affinity and poor protelytic resistance, the peptides should later be tuned up, to obtain more desirable pharmacological properties. Biopanning using bicyclic peptide libraries is now taking the leap from promise to reality, as reflected by the clinical advances from Bicycle therapeutics.

In dealing with low molecular weight ligands, even if proteolysis and affinity issues are circumvented, care must be taken to avoid a rapid kidney excretion before target-binding has occured. Some of the strategies for prolonging the blood half-life of peptides have been reviewed here. However, a short blood half-life will sometimes be advantageous: when using small targeting agents as radioligands, a quick excretion from the system will minimize the unwanted damage produced by the radioligand while it is in the blood.

Certain failed clinical trials of low Mw targeted agents in cancer, such as cilengitide and vintafolide, force us to remain cautious but should not deter the development of this field. The reasons for those flunks at the phase III can only be speculated on. For cilengitide it was suggested to be a poor pharmacokinetics of the peptide, for vintafolide it was suggested to be due to lack of proper patient stratificacion.

Regarding safety considerations on the targeting agents described, to the best of our knowledge, there have been no studies that report immunogenicity. On the contrary, non-immunogenicity was reported for NGR [179] and iRGD [180] in mice. Other positively charged, linear peptides, similar to some of the ones described here, have also shown no immunogenicity in mice [181]. The safety of RGD-based peptides has been confirmed in humans for the case of cilengitide [182], and is currently being evaluated for the case of iRGD (clinical trial identifier: NCT03517176).

In silico mining of peptidic ligands presents itself as an extremely attractive strategy. In light of recent advances in computing power and artificial intelligence, in silico mining could compete with in vitro biopanning for identifying peptidic binders to known proteins, as it is less costly and provides the possibility to fish out peptides with high affinity and high proteolytic resistance [183]. In this context, the progress in techniques for elucidating protein structure, like cryo-electron microscopy [184], and for determining protein structure in a live cell environment, like in-Cell NMR spectroscopy [185], are expected to further accelerate and improve the development of in silico biopanning.

In complex systems like tumors, the variables that affect the conformation, accessibility and dimerization of a target protein (pH, reducing environment, concentration of ions), and hence affect binding, can hardly be reproduced and modeled; for these situations it will still be necessary to use in vivo biopanning. Additionally, in virtual mining a known protein is set as the target, which leaves out many unknown and possibly interesting receptors to target in a tumor. Therefore, we believe that at least in the short term, in silico screening will not displace in vivo biopanning but will synergize with it.

## Figures and Tables

**Table 1:** Docking Protocols suitable for blind peptide docking.

Docking Protocol	Description	URL
AutoDock	A parameter set based on the AMBER force field (Cornell et al. 1995) is used. Flexible or fixed torsions for the ligands can be used	http://autodock.scripps.edu/
HPEPDOCK	Peptide flexibility is included as an ensemble of peptide conformations	http://huanglab.phys.hust.edu.cn/hpepdock/
AnchorDock	Uses prior identification of anchoring spots on the protein surface and the peptide is simulated around the predicted spots.	http://dx.doi.org/10.1016/j.str.2015.03.010
CABS-dock	Multiscale procedure using coarse-grained protein model and a Monte Carlo scheme.	http://biocomp.chem.uw.edu.pl/CABSdock
AutoDock CrankPeP	Folds the peptide in the potential energy landscape created by the receptor.	http://adcp.scripps.edu/
PepVis	Pipeline for automated script generation	https://github.com/inpacdb/PepVis

**Table 2:** Vascular-homing peptides.

Peptide	Sequence/Structure	Ref	Identification	Advantages
**iRGD**	CRGDKGPDC (disulfide cyclized)	[103]	In vitro phage display	* Tumor-penetrating peptide * Medium-high affinity for α_v_β_5_ integrin (K_D_: 62nM) and α_v_β_3_ integrin (K_D_: 18 nM) * Extensively validated in different tumor models * Proven non-immunogenicity in mice * In phase I clinical trials
**Cilengitide**	RGDfV (head to tail cyclized)	[27]	By rational design based on RGD motif	* High affinity for α_v_β_3_ integrin (K_D_: 0.58nM) * Safe in humans
**SFITGv6**	GRCTFRGDLMQLCYPD (head-to-tail cyclized, disulfide bonded)	[119]	In vitro phage display	* High affinity for α_ν_β_6_ integrin (K_D_: 15nM) * Validated applicability in cancer patients
**CNGRC**	CNGRC (disulfide cyclized)	[128]	In vitro phage display	* Validated in vitro as peptide-drug conjugate * Proven non-immungenicity in mice
**RGDechi**	Cyclic RGD/echistatin hybrid PubChem CID: 91936353	[120]	By rational design based on cyclic RGD and echistatin	* Validated applicability in PET imaging * Can discriminate between α_v_β_3_ and α_v_β_5_ integrin

**Table 3:** ECM-homing peptides.

Peptide Name	Sequence/Structure	Ref	Identification	Advantages
**DAG**	CDAGRKQKC (disulfide cyclized)	[20]	In vivo phage display in Alzheimer mice	* Robust homing in patient-derived glioblastoma * Known receptor (CTGF)
**ZD2**	CTVRTSADC (disulfide cyclized)	[139]	In vitro phage display on recombinant protein	* Known receptor and affinity (Fibronectin extra domain B, K_D_:11µM) * PET and MRI active conjugates of ZD2 are being commercially developed by the company Molecular Theranostics
**CSG**	CSGRRSSKC (disulfide cyclized)	[140]	In vitro phage display on Matrigel ^TM^	* Robust homing in different mouse models * Known receptor (laminin-nidogen complex)
**PlGF-2_122-144_**	RRRPKGRGKRRREKQRPTDCHL	[147]	Imitation of natural ligand	* High affinity (low nM K_D_) binding to multiple ECM proteins * Proven therapeutic value
**BT1718 (Peptide-Drug Conjugate)**	Undisclosed (bicyclic peptide coupled to maytansinoid)	[140]	In vitro phage display	* High affinity (low nM K_D_) to MMP-14 * In Phase I/IIa clinical trials

**Table 4:** M2 TAM-targeting agents.

Ligand Name	Sequence	Reference	Identification	Advantages	Disadvantages/Question Marks
**RP-182**	KFRKAFKRFF	[155]	In silico	▪ Proven efficacy in tumor models in mice	▪ Binds to receptors on other cell types ▪ Not reported if conjugation affects binding
**M2pep**	YEQDPWGVKWWY	[156]	In vitro phage display	▪ Proven efficacy in tumor models in mice when conjugated to cytotoxic payload	▪ Must be dissolved in 10% DMSO ▪ Receptor unknown vBinding to human M2s unknown
**cyclic M2pep(RY)**	CGYEQDPWGVRYWYGCkkk	[26]	Rational modification of M2pep	▪ Thoroughly validated specifity to M2 mouse macrophages	▪ Must be dissolved in 10% DMSO ▪ Receptor unknown ▪ Binding to human M2s unknown
**Mannose**		[161]	Natural ligand of CD206	▪ Conjugation does not affect specificity	▪ The receptor is also present in healthy skin and intestine
**mUNO**	CSPGAK	[157,158]	In vivo phage display	▪ Conjugation through its N-terminus and also its Cysteine does not affect specificity ▪ Binds to mouse and human CD206 ▪ Does not compete with mannose ▪ Highly specific to M2 TAMs of different solid tumors in mice	▪ In vivo efficacy unpublished

**Table 5:** Different methods to identify peptidic ligands and their advantages/disadvantages.

Identification	Advantages	Disadvantages/Question Marks
**Phage Display-Cyclic Library (CX_i_C)**	▪ Well validated for in vitro and in vivo biopanning	▪ Affinity and proteolytic resistance generally low
**Phage Display-Bicyclic Library (CX_i_CX_j_C)**	▪ Yields high affinity ligands	▪ Not validated in vivo
**Phage Display-Sunflower Trypsin Inhibitor I Library**	▪ Yields cyclotidic ligand: ▪ high affinity ▪ possible oral activity	▪ Ill-described in the literature
**Mirror Image Phage Display**	▪ Yields peptides with high proteolytic resistance	▪ Can only be performed in vitro or in vivo ▪ Laborious, requires synthesis of D-protein
**Imitation of Natural Binder**	▪ Straightforward	▪ Might bind other receptors in healthy tissue ▪ If a peptide, requires finding the minimal binding motif
**In silico Identification**	▪ Cheaper ▪ Can yield high affinity ligand	▪ Can not probe the protein as is in the disease ▪ Will not identify an unexpected biomarker
